# Genetic variant rs11136000 upregulates clusterin expression and reduces Alzheimer’s disease risk

**DOI:** 10.3389/fnins.2022.926830

**Published:** 2022-08-10

**Authors:** Jin Ma, Shizheng Qiu

**Affiliations:** ^1^Department of Emergency Medicine, Affiliated Kunshan Hospital of Jiangsu University, Kunshan, China; ^2^School of Computer Science and Technology, Harbin Institute of Technology, Harbin, China

**Keywords:** Alzheimer’s disease, genetic variant, *CLU*, genome-wide association study, rs11136000, eQTL

## Abstract

Clusterin (*CLU*) is an extracellular chaperone involved in reducing amyloid beta (Aβ) toxicity and aggregation. Although previous genome-wide association studies (GWAS) have reported a potential protective effect of *CLU* on Alzheimer’s disease (AD) patients, how intron-located rs11136000 (*CLU*) affects AD risk by regulating *CLU* expression remains unknown. In this study, we integrated multiple omics data to construct the regulated pathway of rs11136000-*CLU*-AD. In step 1, we investigated the effects of variant rs11136000 on AD risk with different genders and diagnostic methods using GWAS summary statistics for AD from International Genomics of Alzheimer’s Project (IGAP) and UK Biobank. In step 2, we assessed the regulation of rs11136000 on *CLU* expression in AD brain samples from Mayo clinic and controls from Genotype-Tissue Expression (GTEx). In step 3, we investigated the differential gene/protein expression of *CLU* in AD and controls from four large cohorts. The results showed that rs11136000 T allele reduced AD risk in either clinically diagnosed or proxy AD patients. By using expression quantitative trait loci (eQTL) analysis, rs11136000 variant downregulated *CLU* expression in 13 normal brain tissues, but upregulated *CLU* expression in cerebellum and temporal cortex of AD samples. Importantly, *CLU* was significantly differentially expressed in temporal cortex, dorsolateral prefrontal cortex and anterior prefrontal cortex of AD patients compared with normal controls. Together, rs11136000 may reduce AD risk by regulating *CLU* expression, which may provide important information about the biological mechanism of rs9848497 in AD progress.

## Introduction

Alzheimer’s disease (AD) is a neurodegenerative disease of the central nervous system characterized by progressive cognitive dysfunction and behavioral impairment (Scheltens et al., 2016; Van Cauwenberghe et al., 2016; Pimenova et al., 2018; Hu et al., 2021b). It is estimated that at least 40 million middle-aged and elderly people worldwide suffer from AD (Van Cauwenberghe et al., 2016. Among all the AD susceptibility genes, Apolipoprotein E (*APOE*), which mediates the binding, internalization, and catabolism of lipoprotein particles, is considered to be the major risk factor (Namba et al., 1991; Belloy et al., 2019). *APOE* not only co-deposits with beta-amyloid (Aβ) through protein-protein interaction, but also directly leads to secretion and impaired clearance of Aβ (Namba et al., 1991; Huynh et al., 2017; Belloy et al., 2019).

Other susceptibility genes, such as *CLU*, also affect the occurrence and progression of AD through the accumulation and clearance of Aβ, nerve inflammation, and lipid metabolism (Foster et al., 2019; Uddin et al., 2020a). Previous genome-wide association studies (GWAS) have shown that rs11136000 (*CLU*) is a protective locus for AD risk, and several case-control association studies replicate this result (Harold et al., 2009; Lancaster et al., 2015; Balcar et al., 2021). However, some of other studies report no statistically significant association of rs11136000 on AD or no association in non-European populations (Carrasquillo et al., 2010; Seshadri et al., 2010; Seripa et al., 2018; Zhu et al., 2018). The conflicting results of these studies made us interested in investigating the effect of rs11136000 on AD. Moreover, how rs11136000 regulates *CLU* expression and leads to AD needs further evaluation (Hu et al., 2020, 2021a).

In this study, we integrated multiple omics data, including genome-wide association study (GWAS), expression quantitative trait loci (eQTLs), transcriptome and proteome data, to investigate whether rs11136000 regulates *CLU* expression and thereby contribute to AD. In addition, we identified the different effects of rs11136000 on AD patients of different genders.

## Materials and methods

### Genome-wide association studies datasets

Genome-wide association studies uses single nucleotide polymorphisms (SNPs) in the human genome as molecular genetic markers to analyze the correlation between genotype and phenotype, aiming to discover genetic risk variants that affect phenotype (Tam et al., 2019). A total of five large-scale GWAS datasets for AD were included in the statistical analysis of this study. First, we obtained two GWAS datasets for AD patients with clinical or autopsy diagnosis from International Genomics of Alzheimer’s Project (IGAP), including 17,008 AD cases and 37,154 controls, and 21,982 AD cases and 41,944 controls, respectively (Table 1; Lambert et al., 2013; Kunkle et al., 2019). In addition, we obtained three large GWAS cohorts for AD proxy from UK Biobank, including family history of maternal AD (27,696 cases and 260,980 controls), family history of patrilineal AD (14,338 cases and 245,941 controls), and family history of all AD patients (Marioni et al., 2018). All of the participants were of European descent.

**TABLE 1:** Data sources of GWAS.

Study	Traits	Diagnosis	Cases	Controls	Ethnicity
IGAP2013 (Lambert et al., 2013)	GWAS	Clinical or autopsy	25,580	48,466	European
IGAP2019 (Kunkle et al., 2019)	GWAS	Clinical or autopsy	35,274	59,163	European
UK Biobank (all) (Marioni et al., 2018)	GWAX	Clinical or autopsy	42,034	272,244	European
UK Biobank (maternal) (Marioni et al., 2018)	GWAX	Proxy	27,696	260,980	European
UK Biobank (paternal) (Marioni et al., 2018)	GWAX	Proxy	14,338	245,941	European

GWAX, genome-wide association studies by proxy. GWAS, genome-wide association studies.

### Expression quantitative trait loci datasets

Expression quantitative trait loci are genetic variants that control the expression levels of quantitative trait genes. In particular, variants located in non-coding regions may cause disease by modulating gene expression. In this study, we obtained datasets that rs11136000 regulates gene expression in AD patients and controls, respectively. The eQTL data of AD and non-AD samples were obtained from Mayo clinic and Genotype-Tissue Expression (GTEx) project, respectively (Table 2; Allen et al., 2012; GTEx Consortium., 2017). The Mayo dataset contained gene expression data for temporal cortex (TCX) in 186 AD subjects and 170 normal subjects, and cerebellar tissue (CER) in 191 AD subjects and 181 normal subjects (Allen et al., 2012). In addition, eQTL data of 13 brain tissues, including amygdala, anterior cingulate cortex, caudate, cerebellar hemisphere, cerebellum, cortex, frontal cortex, hippocampus, hypothalamus, nucleus accumbens, putamen, spinal cord, and substantia nigra were obtained from GTEx (version 8) as controls (GTEx Consortium., 2017). The donors in GTEx were of multiple descents including European (85.3%), African (12.3%), Asian (1.4%), etc., (GTEx Consortium., 2017).

**TABLE 2:** The effect of genetic variant rs11136000 on *CLU* expression in AD and normal samples.

Data sources	Brain tissue	No. Samples	Beta	*P*-value
GTEx	Amygdala (non-AD)	88	−0.065	0.23
	Anterior cingulate cortex (non-AD)	109	−0.10	0.027
	Caudate (non-AD)	144	−0.041	0.16
	Cerebellar Hemisphere (non-AD)	125	−0.0012	0.98
	Cerebellum (non-AD)	154	−0.049	0.12
	Cortex (non-AD)	136	−0.058	0.065
	Frontal Cortex (non-AD)	118	−0.068	0.036
	Hippocampus (non-AD)	111	−0.069	0.076
	Hypothalamus (non-AD)	108	−0.0095	0.81
	Nucleus accumbens (non-AD)	130	−0.16	0.00023
	Putamen (non-AD)	111	−0.12	0.00082
	Spinal cord (non-AD)	83	−0.064	0.24
	Substantia nigra (non-AD)	80	−0.021	0.75
MAYO	Cerebellum (AD)	186	0.0635	0.23
	Cerebellum (non-AD)	170	−0.0905	0.048
	Temporal cortex (AD)	191	0.0588	0.031
	Temporal cortex (non-AD)	181	0.286	0.00029

Beta is the regression coefficient based on the effect allele. Beta > 0 and beta < 0 mean that this effect allele could increase and reduce gene expression, respectively. The statistically significant association is defined to be P < 0.05/17 = 0.00294.

### RNA expression datasets

RNA-seq data for AD versus controls was generated from over 2,100 samples from post-mortem brains of more than 1,100 individuals from seven distinct brain regions from three human cohort studies, including Religious Orders Study and Memory and Aging Project (ROSMAP), Mayo RNAseq (MAYO), and Mount Sinai Brain Bank (MSBB) (Bennett et al., 2012a,b; Zou et al., 2012; Ng et al., 2017). The seven brain regions contained dorsolateral prefrontal cortex (DLPFC), CER, TCX, frontal pole (FP), inferior frontal gyrus (IFG), parahippocampal gyrus (PHG), and superior temporal gyrus (STG).

### Proteomics datasets

Proteomic data was generated from post-mortem brains of more than 500 individuals from four human cohort studies, including Banner Sun Health Research Institute (Banner), Baltimore Longitudinal Study on Aging (BLSA), MAYO and MSBB. Brain samples consisted of four different brain regions [DLPFC, Middle Frontal Gyrus (MFG), TCX and Anterior Prefrontal Cortex (AntPFC)]. Protein abundance was quantified using liquid-free quantification (LFQ). The proteomic data was adjusted for age, sex, and post mortem interval (PMI).

### The effect of genetic variant rs11136000 on Alzheimer’s disease risk

We investigated the effect of rs11136000 T allele on AD risk in GWAS summary statistics for AD of clinically diagnosed or autopsy and first-degree relative proxies, respectively. In addition, we explored the effect of rs11136000 on AD patients with different genders using GWAS by proxy (GWAX) from UK Biobank. The statistically significant association is defined to be *P* < 5E-08 after adjusting for multiple testing.

### The effect of rs11136000 on clusterin expression in Alzheimer’s disease and controls

We investigated the potential differential *cis*-regulated effect of rs11136000 on *CLU* in AD versus controls using an additive model eQTL analysis (Hu et al., 2020, 2021b; Qiu et al., 2022). According to the additive model, each allele has an independent effect on the trait. Here, we coded the possible genotypes of rs11136000 (TT = 2, TC = 1, CC = 0), where T is an effect allele and C is a non-effect allele. Thus, the differential regulation of *CLU* expression in rs11136000 T allele carriers of AD and controls can be calculated using linear regression models. The statistically significant association is defined to be *P* < 0.05/(number of brain tissues) = 0.05/17 = 0.00294 after multiple testing.

### Differential expression of clusterin between Alzheimer’s disease and normal individuals

We evaluated the differential mRNA expression of *CLU* in seven brain regions between AD and controls from ROSMAP, MAYO and MSBB. Meanwhile, we investigated the differential protein expression of *CLU* in four brain regions of AD versus controls from DLPFC, MFG, TCX, and AntPFC. The differential expression was determined via ANOVA. The significance level of differential expression was defined as *P* < 0.05/7 = 0.00714 and *P* < 0.05/4 = 0.0125 after multiple testing.

## Results

### rs11136000 T allele reduced Alzheimer’s disease risk

**FIGURE 1:**
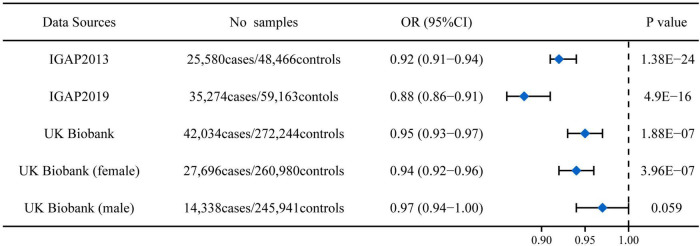
Association between rs11136000 variant T allele and AD. IGAP, International Genomics of Alzheimer’s Project. The statistically significant association is defined to be *P* < 5E-08.

rs11136000 T allele significantly reduced AD risk in both clinically diagnosed Alzheimer’s cohorts from IGAP (OR: 0.92, 95%CI: 0.91-0.94, *P* = 1.38E-24; OR: 0.88, 95%CI: 0.86-0.91, *P* = 4.90E-16) (Figure 1). In the UK Biobank cohort (using participants whose parents suffered from AD as a proxy for cases), rs11136000 T allele was suggestively protective against AD (OR: 0.95, 95%CI: 0.93-0.97, *P* = 1.88E-07) (Figure 1). However, rs11136000 only potentially affected female individuals with AD (OR: 0.94, 95%CI: 0.92-0.96, *P* = 3.96E-07).

### rs11136000 upregulated clusterin expression in Alzheimer’s disease

rs11136000 downregulated *CLU* expression in all 13 normal brain tissues from GTEx, two of which passed multiple testing (*P*_*nucleusaccumbens*_ = 0.00023 and *P_*putamen*_* = 0.00082) (Table 2). However, rs11136000 suggestively upregulated *CLU* expression in cerebellum (β = 0.0635, *P* = 0.23) and temporal cortex samples of AD (β = 0.0588, *P* = 0.031) (Table 2).

### Clusterin differentially expressed in Alzheimer’s disease versus controls

**FIGURE 2:**
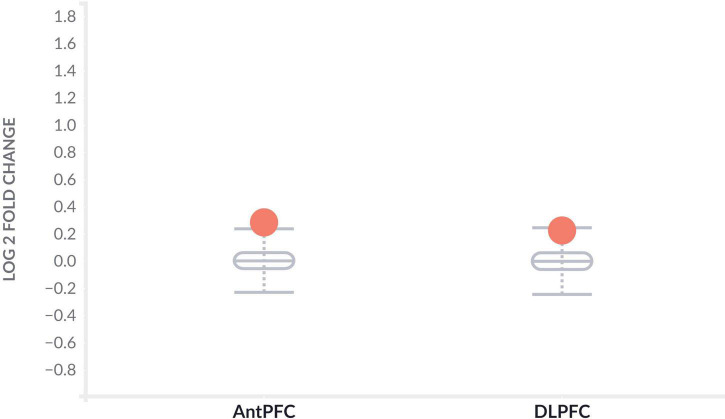
Differential protein expression of *CLU* between AD and normal samples. The gray boxplots represent the expression levels of *CLU* protein in the brain tissues of healthy participants. The orange dots represent the expression levels of *CLU* protein in the brain tissues of AD patients. AntPFC, anterior prefrontal cortex; DLPFC, dorsolateral prefrontal cortex.

To further determine the effect of *CLU* in AD patients, we investigated the differential expression of *CLU* between AD and controls in various brain regions at the level of gene expression and protein expression, respectively. The *CLU* mRNA expression in temporal cortex region of AD patients significantly differed from controls regardless of gender (${\text{log}}_{2}^{F\,C}$ = 0.83, *P*_*TCX*_ = 2.96E-10) (Table 3). However, *CLU* was only significantly differentially expressed in parahippocampal gyrus region of female AD patients compared to controls (${\text{log}}_{2}^{F\,C}$ = 0.34, *P*_*PHG*_ = 0.00032). Furthermore, *CLU* protein was detected in DLPFC and AntPFC, and was significantly differentially expressed in both two brain tissues (${\text{log}}_{2}^{F\,C}$ = 0.29, *P*_*AntPFC*_ = 0.00022; ${\text{log}}_{2}^{F\,C}$ = 0.23, *P*_*DLPFC*_ = 5.09E-06) (Figure 2).

**TABLE 3:** Differential mRNA expression of *CLU* in AD and normal samples.

Phenotype	Brain tissue	$\text{l}\,o\,g_{2}^{F\,C}$	*P*-value
AD (all)	Cerebellum	0.12	0.43
	Dorsolateral Prefrontal Cortex	0.016	0.78
	Frontal Pole	0.15	0.033
	Inferior Frontal Gyrus	0.13	0.13
	Parahippocampal Gyrus	0.31	8.17E-07
	Superior Temporal Gyrus	0.17	0.27
	Temporal Cortex	0.83	2.96E-10
AD (female)	Cerebellum	0.20	0.29
	Dorsolateral Prefrontal Cortex	−0.013	0.87
	Frontal Pole	0.15	0.23
	Inferior Frontal Gyrus	0.19	0.14
	Parahippocampal Gyrus	0.34	0.00032
	Superior Temporal Gyrus	0.23	0.061
	Temporal Cortex	0.82	1.89E-06
AD (male)	Cerebellum	0.012	0.97
	Dorsolateral Prefrontal Cortex	0.028	0.78
	Frontal Pole	0.12	0.38
	Inferior Frontal Gyrus	0.64	0.79
	Parahippocampal Gyrus	0.24	0.084
	Superior Temporal Gyrus	0.076	0.67
	Temporal Cortex	0.84	0.00004

${\text{log}}_{2}^{F\,C}$: log fold change value.

## Discussion

Large-scale GWAS in recent years have identified substantial genetic variants and genes associated with AD risk (Jansen et al., 2019; Kunkle et al., 2019; Schwartzentruber et al., 2021). Susceptibility loci including *APOE* have been confirmed by numerous studies (Al Mamun et al., 2020; Uddin et al., 2020b). *CLU*, also known as apolipoprotein J (APOJ) protein, is identified as the third-highest risk gene for late-onset AD (LOAD), contributing approximately 9% of AD risk (Bertram et al., 2007; Foster et al., 2019; Uddin et al., 2020a). Previous studies have shown that elevated *CLU* levels have been detected in the brain and plasma of AD individuals and are involved in neuroinflammation, lipid metabolism, and Aβ clearance in AD patients (Lidstrom et al., 1998; Bu, 2009; Thambisetty et al., 2010; Uddin et al., 2020a). However, some studies have also reported that *CLU* incorporation into amyloid aggregates is more harmful than Aβ42 aggregates alone (Uddin et al., 2020a). The ambiguous and complex role of *CLU* in AD prevents it from becoming a therapeutic target for AD.

In this study, we integrated GWAS, eQTL, gene expression and protein expression data to investigate whether rs11136000 (*CLU*) affects AD risk by regulating *CLU* expression. We successfully explained the pathway of rs11136000-CLU-AD. The results showed that rs11136000 significantly reduced AD risk in both clinically diagnosed AD and AD proxy. The effects of rs11136000 on AD risk with different genders and different diagnostic modalities were slightly different. In addition, previous meta-analyses and systematic reviews suggested that the heterogeneity of rs11136000 on AD risk was also reflected by race. Both Han et al. (2018) and Zhu et al. (2018) believed that rs11136000 only reduced the risk of AD in the European population, while the association was weak in the East Asian population. Subsequent eQTL analysis revealed heterogeneity of rs11136000 expression in various brain tissues. Significant difference of the regulation of rs11136000 on *CLU* expression was only showed in temporal cortex region between AD patients versus controls. Interestingly, *CLU*-immunopositive Aβ deposits were found in the temporal cortex of AD patients, and 29% of Aβ in brain tissue was associated with *CLU* protein (Martin-Rehrmann et al., 2005; Uddin et al., 2020a).

The study has some advantages. The multiple omics data used in this study were all from European populations, avoiding the bias associated with population stratification. Multiple omics data constructed a complete pathway that genetic variants regulate gene expression and then affect disease phenotype, which better explains the role of rs11136000 in the brain of AD patients than previous studies. However, this study also has certain limitations. It is difficult for us to obtain gender- and ethnic-specific multi-omics data, which limits the further disclosure of the specific regulatory role of rs11136000 on AD patients in different populations.

In conclusion, this study highlights the potential role of the variant rs11136000 on AD risk by regulating *CLU* expression. These findings reveal the importance of a better understanding of *CLU* function and dysfunction in the context of normal and AD individuals.

## Data availability statement

The original contributions presented in this study are included in the article/supplementary material, further inquiries can be directed to the corresponding author.

## Author contributions

SQ analyzed the data and drafted the manuscript. JM designed the study, revised the manuscript, and supervised this work. Both authors reviewed and approved the final version.
